# Itaconic Acid Directly Binds to the Cysteine Residue of PurF: A Novel Mechanism for Inhibiting Purine Metabolism in Porcine Pathogenic *Escherichia coli*

**DOI:** 10.3390/microorganisms14081852

**Published:** 2026-08-20

**Authors:** Haozhen Liu, Xin Li, Xinyu Zhang, Yao Ge, Yinfeng Chen, Xinjian Li, Zhenlong Wu

**Affiliations:** 1 State Key Laboratory of Animal Nutrition and Feeding, China Agricultural University, Beijing 100193, China; 2 State Key Laboratory of Epigenetic Regulation and Intervention, Institute of Biophysics, Chinese Academy of Sciences, Beijing 100101, China; 3 Beijing Advanced Innovation Center for Food Nutrition and Human Health, China Agricultural University, Beijing 100193, China

**Keywords:** itaconic acid, purine metabolism, PRPP amidotransferase (PurF), porcine extraintestinal pathogenic *Escherichia coli* (PCN033)

## Abstract

Itaconic acid has been reported to possess anti-inflammatory and antibacterial properties. However, its specific mechanisms of action against pathogenic bacteria, especially in the context of purine metabolism, remain poorly understood. Here, we investigate the impact of itaconic acid on the purine metabolism of PCN033, a highly pathogenic porcine extraintestinal pathogenic *Escherichia coli* (ExPEC) strain. Our in vitro and in vivo experiments demonstrated that itaconic acid significantly inhibited the proliferation of PCN033. Multi-omics analyses, including transcriptome and metabolome sequencing, revealed that itaconic acid severely disrupted the purine metabolism pathway of PCN033. Further mechanistic studies identified PRPP amidotransferase (PurF), a key enzyme in de novo purine synthesis, as a direct target of itaconic acid. Molecular docking and click chemistry experiments provided compelling evidence that itaconic acid specifically binds to the second cysteine residue (2C) of PurF, leading to the inhibition of its enzymatic activity. These data reveal a novel and critical mechanism by which itaconic acid exerts its antibacterial effects in response to bacterial infection. Importantly, our in vivo data show that supplementation with itaconic acid alleviated weight loss, organ damage, and inflammatory responses induced by PCN033 infection in mice and nursery pigs. These novel findings enhance our understanding of the antibacterial mechanisms of itaconic acid. Supplementation with itaconic acid could serve as a therapeutic strategy for treating pathogenic bacterial infections in humans and other animals.

## 1. Introduction

Extra-intestinal pathogenic *Escherichia coli* (ExPEC) is one of the main zoonotic pathogens responsible for multiple infections in non-intestinal locations in a broad range of animals, such as humans, birds, pigs, and avian species, posing a great threat to public health worldwide [1,2]. It has been reported that virulence factors play an important role in the diverse ExPEC pathotypes. However, the underlying mechanisms of ExPEC’s ability to break through the barriers and evade immune clearance remain largely unknown [3]. Moreover, ExPEC strains with potent resistance to antibiotics, including cephalosporins and fluoroquinolones, have been identified, highlighting an impediment to therapeutic options for ExPEC-related infections in humans and other animals [4].

PCN033 is a porcine-derived ExPEC strain that was isolated from diseased pigs by Professor Huanchun Chen at Huazhong Agricultural University between 2006 and 2008 [5]. Infection with ExPEC PCN033 leads to meningitis and sepsis in pigs [6,7]. It has been reported that virulence plasmids and a functional type VI secretion system (T6SS) of PCN033 are associated with the pathogenesis and pathogenic signs in animals [8,9,10]. Additionally, adhesion factors, specific capsular polysaccharides, and iron acquisition systems have been reported to be critical players in terms of survival capacity in the host’s micro-environment [6,11,12]. Importantly, the presence of drug-resistance-related genes in the porcine-derived strain has led to its use as a special model for investigating potential drugs for treating ExPEC-related disorders owing to the similarity between human- and porcine-derived ExPEC.

Bacterial purine homeostasis is crucial for the survival and proliferation of various microorganisms, including human- and porcine-derived ExPEC [13], because of a crucial effect purine metabolism has on the synthesis of nucleotides and the generation of energy carriers (ATP/GTP) and cofactors, thereby benefitting the proliferation and survival of pathogenic bacteria [2]. ExPEC has conserved purine biosynthesis routes, including de novo and salvage synthesis. Notably, de novo purine biosynthesis is one of the fundamental metabolic processes through which bacteria utilize precursor molecules, such as phosphoribosyl pyrophosphate (PRPP), glutamine, aspartate, and glycine, to produce purines and purine nucleotides to support bacterial growth. This pathway is metabolically costly (requiring 6 ATP molecules per molecule of purine synthesized) and regulated at the transcriptional and post-transcriptional levels [14]. Among the enzymes implicated in de novo purine synthesis is PRPP amidotransferase (encoded by PurF), a limiting enzyme. Consistently, deletion of purF has been reported to disrupt purine synthesis, thereby inhibiting *Escherichia coli* proliferation, reducing synthesis and secretion of virulence factors, inducing susceptibility to antibiotics, and impairing bacterial pathogenicity [15,16,17,18]. In addition, *E. coli* also possesses an efficient salvage pathway that reutilizes preformed purines from exogenous bases or nucleosides to produce purines in an energy-efficient manner in response to nutrient deprivation. Considering nucleotides’ critical role in genetic information transfer, energy metabolism, cofactor biosynthesis, and cellular communication, targeting de novo purine biosynthesis could be a therapeutic strategy with which to improve the health of humans and other animals.

Itaconic acid, the product of the decarboxylation of cis-aconitate, is predominantly biosynthesized in activated macrophages via immune-responsive gene 1 (IRG1)-mediated pathways [19]. Studies have revealed that itaconic acid has numerous bioactivities, such as regulation of fatty acid β-oxidation, generation of mitochondrial reactive oxygen species, metabolic reprogramming, polarization regulation, inhibition of cytokine production, and regulation of oxidative stress [19,20,21]. Studies have also demonstrated that itaconic acid secreted by macrophages can be exported from the cytosol to the extracellular space with the help of ATP-binding cassette transporter G2 (ABCG2), indicating a regulatory effect on other cells in various biological processes [22]. Consistently, itaconic acid and its derivative 4-octyl itaconate (4-OI, a cell-penetrating derivative of itaconic acid) have exhibited health benefits in the treatment of inflammatory diseases, tumors, and microbial infections [23,24,25]. Despite these advances in itaconic acid research under various conditions, it remains unknown whether it can alleviate ExPEC-infection-induced intestinal barrier dysfunction in humans and animals. In this study, using both in vitro and in vivo models, we investigated the impact of itaconic acid supplementation on intestinal barrier dysfunction induced by PCN033—a highly pathogenic porcine extraintestinal pathogenic *Escherichia coli* (ExPEC strain—and the underlying mechanism related to purine metabolism.

## 2. Materials and Methods

All experiments were performed in accordance with the ARRIVE (Animal Research: Reporting of In Vivo Experiments) and RIGOR guidelines and are reported accordingly. The animal procedures were approved by the Laboratory Animal Welfare and Animal Experimental Ethical Committee of China Agricultural University (Approval No. AW32504202-1-3). Anesthesia was induced by intraperitoneal injection of sodium pentobarbital (50 mg/kg). Euthanasia was performed in accordance with the 2020 AVMA Guidelines for the Euthanasia of Animals. For mice, euthanasia was achieved via cervical dislocation following deep anesthesia. For piglets, euthanasia was performed via intravenous injection of an overdose of sodium pentobarbital (100 mg/kg) to ensure humane and rapid loss of consciousness and cessation of vital functions. The pigs were confirmed dead via monitoring heartbeat, respiration, and pedal reflexes. Animals that died or were euthanized prior to the endpoint were excluded from the analysis. Three or more independent biological replicates were performed for all experiments. Treatment administration, data collection, and analysis were performed in a blinded manner by employing different investigators or masking sample labels.

### 2.1. Animals and Housing

The IRG1 knockout (IRG1^−/−^) mice used in this study were a gift from Xinjian Li’s team. Male C57BL/6 mice (11 ± 1 g) were purchased from Beijing HFK Biotechnology Co., Ltd. (Beijing, China). Six-week-old male post-weaning piglets (10 ± 1.5 Kg) were purchased from Beijing HFK Biotechnology Co., Ltd. (Beijing, China). Those with no history of illness and within a normal weight range were selected for this study.

### 2.2. Source of the Fecal Samples and Strain Background

Fecal samples were obtained from a commercial swine farm experiencing an outbreak of a bacterial infection. The pathogenic strain PCN033 used in this study was originally isolated from this farm. Owing to commercial confidentiality agreements with the farm, specific epidemiological data cannot be disclosed.

### 2.3. Donor Selection for Fecal Microbiota Transplantation (FMT)

To prepare for FMT, diarrheic fecal samples were collected from early-weaned piglets (21 days of age) during the outbreak. Diarrheic piglets were strictly defined as those exhibiting liquid, watery feces for at least two consecutive days without any prior antibiotic treatment. Healthy piglets, serving as the control FMT donors, were defined as those with no history of diarrhea or other clinical illnesses. In total, 55 healthy and 45 diarrheic piglets were randomly selected. Fresh fecal samples were collected, aliquoted, and either used immediately for FMT or snap-frozen and stored at −80 °C for future use.

### 2.4. Preparation of Suspensions for Fecal Microbiota Transplantation (FMT)

For FMT, fresh fecal samples from donors with identical health statuses were pooled and processed under anaerobic conditions. Samples were homogenized and diluted 5-fold in sterile PBS (0.1 M, pH 7.2) containing 15% (*v*/*v*) glycerol. The suspension was centrifuged at 100× *g* for 2 min. The supernatant was filtered through a 70 μm cell strainer. For supernatant sterile fecal filtrate (SFF) preparation, the filtered supernatant was further passed through 0.22 μm filters. Final suspensions were aliquoted into cryotubes and stored at −80 °C until use.

### 2.5. Design of the Experiments

Experiment 1: Four-week-old male C57BL/6 mice (c) were subjected to a 7-day broad-spectrum antibiotic cocktail treatment (penicillin, 0.5 g/L; streptomycin, 0.5 g/L; vancomycin, 0.5 g/L; and metronidazole, 0.25 g/L) in drinking water to deplete the gut microbiota. The mice were then randomly divided by weight into two groups: the control group received oral gavage of a healthy pig fecal suspension (10^8^ CFU/mL, 0.1 mL), and the treatment group received oral gavage of a diarrheic pig fecal suspension (10^8^ CFU/mL, 0.1 mL). Gavage was performed every other day. Frozen suspensions were thawed at 37 °C and mixed thoroughly before each administration. At the experimental endpoint, all mice were euthanized, and blood, intestinal segments, and fecal samples were collected.

Experiment 2: Four-week-old male IRG1^−/−^ mice (11 ± 1 g) were randomly divided by weight into three groups: (1) an IRG1^−/−^ control (oral gavage of 0.2 mL of saline every other day); (2) IRG1^−/−^ + FMT (oral gavage of 0.1 mL of diarrheic pig fecal suspension (10^8^ CFU/mL) supplemented with 0.1 mL of saline every other day to maintain equal volume); and (3) IRG1^−/−^ + FMT + ITA (administration of itaconate (ITA) via oral gavage at a dose of 10 mg/kg/day plus oral gavage of diarrheic pig fecal suspension as performed in the IRG1^−/−^ + FMT group). Fecal suspensions were prepared as described above. At the experimental endpoint, all mice were euthanized, and samples were collected for analysis.

Experiment 3: Six-week-old male post-weaning piglets (10 ± 1.5 kg) were purchased from a commercial breeding facility (Beijing HFK Biotechnology Co., Ltd., Beijing, China) three days after weaning and acclimatized for 3 days. The pigs were then randomly assigned to five groups: (1) control (CON); (2) ITA (itaconate at 10 mg/kg/day in feed); (3) PCN033.ΔpurF (low-virulence challenge (LV)); (4) PCN033.ΔpurF + ITA (LV + ITA); and (5) PCN033 (high-virulence challenge (HV)). The pigs in the challenge groups were intraperitoneally injected every three days with either the attenuated *E. coli* strain PCN033.ΔpurF (LV and LV + ITA groups) or PCN033 (HV group) at 10^8^ CFU/mL for a total of 10 mL. The strains were cultured in fresh LB medium for 12 h prior to each challenge. Itaconate was dissolved in deionized water and mixed into feed for the respective groups. All groups were fed twice daily, with each meal providing feed at 2% of the animals’ bodyweights. At the experimental endpoint, all pigs were euthanized, and samples were collected.

### 2.6. Bacterial Strains and Culture

The porcine extraintestinal pathogenic *E. coli* strain PCN033 (isolated from diseased pig brain tissue) and its knockout strain PCN033.ΔpurF were used. The strains were routinely cultured aerobically in Luria–Bertani (LB) broth at 37 °C with shaking at 220 rpm or on LB agar plates at 37 °C. For selection of PCN033.ΔpurF, chloromycin was added to the agar at 0.25 µg/mL.

### 2.7. Cell Culture

The Raw264.7 macrophage cell line (ATCC, TIB-71) was cultured in DMEM (Thermo Fisher Scientific, Waltham, MA, USA, C11995500CP) supplemented with 10% FBS (Gibco, Waltham, MA, USA, 10099-141C) and 1% penicillin–streptomycin (Gibco, 15140122) at 37 °C in a 5% CO_2_ incubator.

### 2.8. Intestinal Microbiota Analysis

16S rRNA Gene Sequencing: Total genomic DNA was extracted from fecal samples using the QIAamp Fast DNA Stool Mini Kit (Qiagen, Hilden, Germany). The V3-V4 region of the bacterial 16S rRNA gene was amplified and sequenced using the Illumina MiSeq platform (Illumina, San Diego, CA, USA). After demultiplexing and quality filtering, sequences were analyzed using the QIIME2 pipeline. Denoising was performed with DADA2, and taxonomy was assigned against the SILVA132 database using a naïve Bayes classifier.

### 2.9. Gene Knockout

In this study, the complete genome sequence of the strain PCN033 was retrieved from the NCBI database. The purF gene was identified, and 500 bp upstream and downstream flanking fragments were selected as the upstream and downstream homology arms for gene knockout. Verification primers were designed for the purF locus. Primers for the chloramphenicol resistance gene (cmr) were designed using pKD3 (Miaoling P0095) as the template. The upstream homology arm, cmr cassette, and downstream homology arm were fused into a single linear fragment via overlap PCR.

The fused fragment was cloned into the suicide plasmid pCVD442 (Miaoling P8527). The recombinant pCVD442 was transformed into the auxotrophic *Escherichia coli* strain WM3064 (BiosciC1039). Conjugation was performed between PCN033 and WM3064 in vitro. Transconjugants were selected on LB agar plates supplemented with chloramphenicol but without diaminopimelic acid. Single colonies were chosen and verified by PCR and sequencing.

Positive colonies were further cultured and then streaked on LB plates containing 10% sucrose and chloramphenicol to select for double-crossover recombinants. Finally, the establishment of the purF knockout mutant was confirmed via PCR amplification and DNA sequencing.

### 2.10. Protein Expression and Mutant Construction

For protein expression, the purF coding region was amplified using specific primers. The pET-28a vector (Miaoling P0023) and the purF fragment were digested with the restriction enzymes SphI (NEB R3182V) and SacI (NEB R3156V). The purF gene was ligated into pET-28a via homologous recombination. The recombinant pET-28a-purF plasmid was electroporated into *E. coli* BL21(DE3).

Protein expression was induced by applying 0.1 mM IPTG for 8 h. Site-directed mutagenesis of purF (2C, 42C, and 459C variants) was performed using a mutagenesis kit, and the mutant proteins were expressed using the same procedure.

### 2.11. Purification of PurF Protein and Mutants

The expression-induced *E. coli* samples (wet weight: ~5 g) were resuspended in 20 mL lysis buffer (50 mM Tris-HCl pH 8.0, 1 mM EDTA, 1 mM PMSF) and sonicated on ice (400 W, 3 s on/5 s off, 10 min total). After centrifugation (12,000× *g*, 4 °C, 20 min), the supernatant was discarded, and the sample was washed twice with 20 mL wash buffer (20 mM Tris-HCl pH 8.0, 1 M EDTA, 2 M urea, 1% Triton X-100) via gentle shaking at room temperature for 15 min followed by centrifugation (12,000× *g*, 4 °C, 20 min) until the precipitate appeared milky white. The washed inclusion bodies were dissolved in 8 M urea and centrifuged (12,000× *g*, 4 °C, 30 min), and the supernatant was collected. The denatured protein was renatured via slow addition to chilled L-arginine renaturation buffer, followed by incubation at 4 °C for 12–16 h. After centrifugation (12,000× *g*, 4 °C, 20 min), the supernatant was dialyzed against 4 L dialysis buffer (20 mM Tris-HCl pH 8.0, 150 mM NaCl) at 4 °C using a 3.5–10 kDa MWCO membrane (three exchanges: 4 h first, followed by 8 h each for 20 h total). The dialyzed solution was filtered through a 0.22 μm membrane, assessed for purity via SDS-PAGE, and stored at –80 °C in aliquots.

The following primers were used in this study:

PURF.2C-F: GAATTCATGgccGGTATTGTCGGTATCGCCGGTG.

PURF.2C-R: GACAATACCggcCATGAATTCGGATCCGCGACCC.

PURF.459C-F: AGTTTGAGgccTCGGTGTTCAACGGCGTTTACGT.

PURF.459C-2R: GAACACCGAggcCTCAAACTGCTGAATATCCGGGTTTTCAGC.

PURF.42C-2F: CCAATAACgccTTCCGTTTGCGTAAAGCGAACGG.

PURF.42C-2R: AACGGAAggcGTTATTGGCATCTATGGTGATGATGCCGG.

purF-up-SacI-F: gtggaattcccgggagagctcATGGTGTTTCACGCACGCAGAG.

purF-up-R: CGGGGCGTAAAACGAAGGATGATGCCTTCGCTCA.

CMR-PURF-F: CATCCTTCGTTTTACGCCCCGCCCTGCCA.

CMR-PURF-R: TATTGTCGGTtgatcggcacgtaagaggttccaactt.

purF-down-F: gtgccgatcaacCGACAATACCGCACATACGTCTT.

PURF-down-SphI-R: tcttctagaggtaccgcatgcTACGCCATAATCGCGGTGATTGCT.

PURF-veri-F: GCAGACTTTGCAGGATTTCGGCG.

purF-veri-R: CCAGAGCGTAAGTGCTCTGAGATGT.

CMR-PURF-2R: GTGCGGTATTGTCGGTtgatcggcacgtaagaggttccaactt.

CMR-PURF-2F: ggcatCATCCTTCGTTTTACGCCCCGCCCTGCCA.

PURF-exp-R: gtggtggtggtggtgctcgagTCCTTCGTTATGCATTTCGAGATTTTCCAC.

PURF-exp-F: atgggtcgcggatccgaattcATGTGCGGTATTGTCGGTATCG.

Site-Directed Mutagenesis: Point mutations (2C, 42C, and 459C) in PurF were introduced using a site-directed mutagenesis kit, with primers listed in the text. Mutant proteins were expressed and purified in a manner similar to that used for the wild type.

### 2.12. Click Chemistry for Itaconate–Protein Interaction

BL21(DE3) cell lysate (200 µL) was incubated with 100 µM Itaconate-alkyne (MCE, HY-133870) at room temperature for 60 min. Subsequently, 200 µM Azide-Rhodamine (Sigma, Livonia, MI, USA, 760757), 1 mM CuSO_4_·5H_2_O, 2.5 mM TBTA ligand (MCE, HY-116677), and 5 mM sodium ascorbate were added. The reaction mixture was incubated in the dark at room temperature for 2 h and then subjected to SDS-PAGE analysis.

### 2.13. Immunofluorescence and TUNEL Staining

Immunofluorescence was performed as previously described. Image analysis was conducted using Image Pro Plus v.6.0 software. Apoptosis in intestinal tissue sections was detected using the TUNEL method with an In Situ Cell-Death Detection Kit (Roche, Basel, Switzerland) in accordance with the manufacturer’s instructions.

### 2.14. Western Blotting

Proteins were extracted from intestinal tissues using RIPA lysis buffer (Roche) and quantified with a BCA assay kit (Pierce). The proteins were separated via SDS-PAGE, transferred to nitrocellulose membranes (Pall), and blocked for 2 h. Membranes were incubated overnight with primary antibodies followed by appropriate HRP-conjugated secondary antibodies. Signals were detected using a chemiluminescence imaging system (Bio-Rad). The following antibodies were used: BAX (Proteintech, Rosemont, IL, USA, 50599-2-Ig), BCL2 (Proteintech, 12789-1-AP), GAPDH (Proteintech, 60004-1-Ig), CASP3 (Proteintech, 19677-1-AP), Cleaved CASP3 (Abcam, Cambridge, MA, USA, ab2302), HO-1 (Proteintech, 10701-1-AP), NQO1 (Proteintech, 11451-1-AP), NRF2 (Abcam, ab137550), OCCLUDIN (Abcam, ab216327), SLC1A5 (Proteintech, 12332-1-AP), SLC13A3 (Proteintech, 17785-1-AP), GSS (Abcam, ab189034), and GLS (Proteintech, 19959-1-AP).

### 2.15. Statistical Analysis

Data are presented as means ± SEMs. Comparisons between two groups were analyzed using Student’s *t*-test. Comparisons between more than two groups were analyzed via one-way ANOVA followed by Tukey’s post hoc test. A *p*-value < 0.05 was considered statistically significant. The specific number of replicates (n) for each experiment is indicated in the figure legends.

## 3. Results

### 3.1. Experiment 1: Oral Administration of Bacteria from Diarrheic Pig Feces to Mice Caused a Series of Adverse Effects

**Figure 1:**
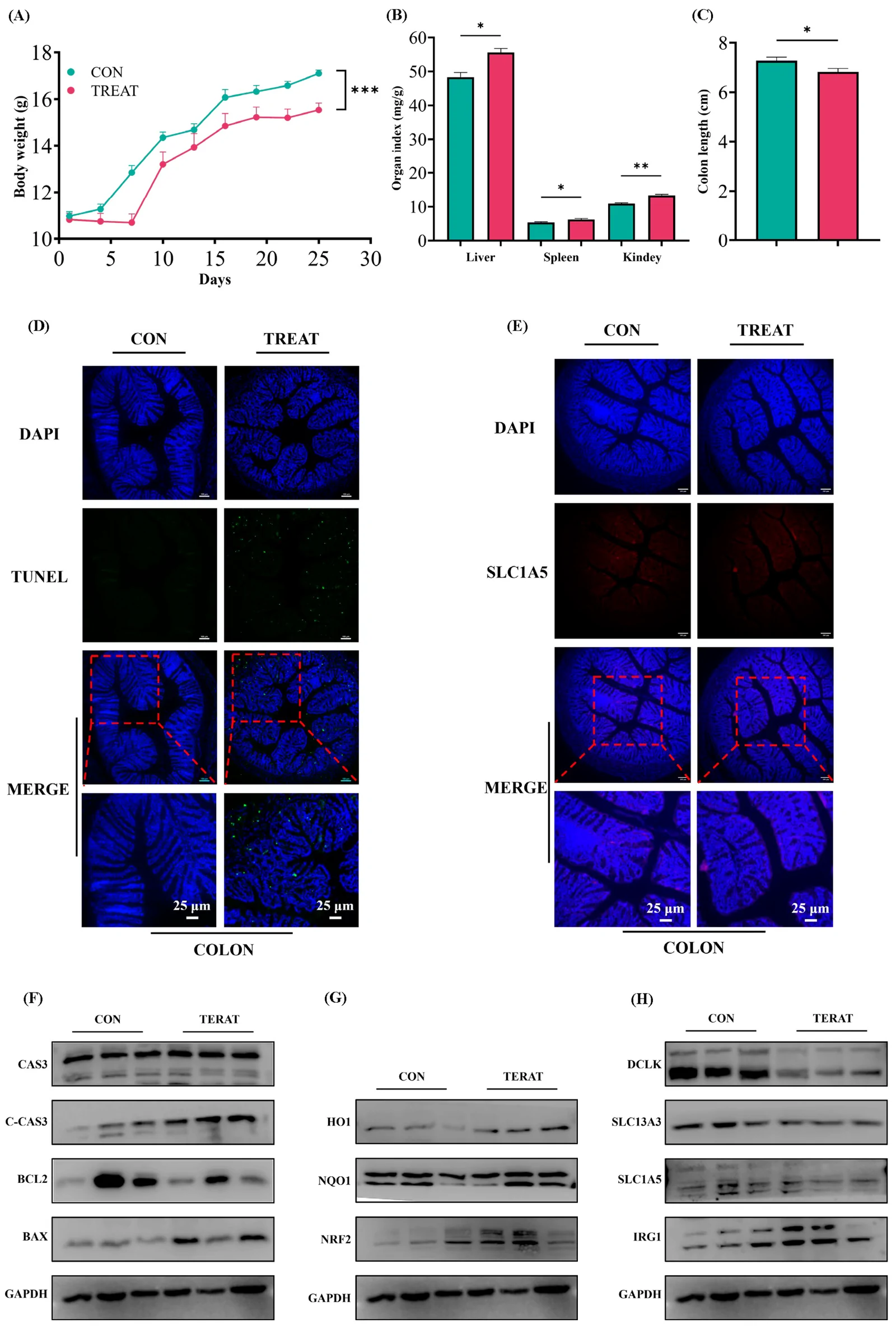
Oral administration of bacteria derived from diarrheic pig feces to mice had a series of adverse effects. (**A**) Daily weight gain of mice in the control group (gavaged with healthy pig feces) and treatment group (gavaged with diarrheic pig feces). (**B**) Organ indices (liver, spleen, and kidney) of mice in the control and treatment groups. The organ index was calculated as follows: (organ weight/bodyweight). (**C**) Length of colons of mice in the control and treatment groups. Colon samples were collected after sacrifice, and length was measured with a vernier caliper. (**D**) Immunofluorescence staining of cell apoptosis in the colon tissues of mice in the control and treatment groups. Tissues were stained with TUNEL reagent (green, apoptotic cells) and DAPI (blue, nuclear staining). Scale bar = 100 μm. (**E**) Immunofluorescence staining of SLC1A5 protein in the colon tissues of mice in the control and treatment groups. SLC1A5 was labeled with a specific primary antibody and a Cy3-conjugated secondary antibody (red), and nuclei were stained with DAPI (blue). Scale bar = 100 μm. The mean fluorescence intensity (MFI) of SLC1A5 was quantified using ImageJ (version 1.54i) software. (**F**) Expression of apoptosis-related proteins (BAX, BCL-2, and cleaved CASPASE-3) in the colon tissues of mice in the control and treatment groups. (**G**) Expression of NRF2-pathway-related proteins in the colon tissues of mice in the control and treatment groups. (**H**) Expression of itaconic-acid-related proteins and amino acid transporter proteins in the colon tissues of mice in the control and treatment groups. All data are presented as means ± SEMs (n = 12 mice per group for (**A**–**C**); n = 6 biological replicates per group, with 5 fields per section for (**D**,**E**); n = 6 biological replicates per group for (**F**–**H**). Statistical significance was determined using an unpaired two-tailed Student’s *t*-test. * *p* < 0.05, ** *p* < 0.01 and *** *p* < 0.005 vs. the control group.

Mice purchased from Huafukang Biological Co., Ltd., were subjected to antibiotic clearance (penicillin, streptomycin, vancomycin, and metronidazole) while being fed normally. After one week of adaptation, the mice were randomly divided into control and treatment groups based on bodyweight and fecal suspensions prepared from a commercial swine farm were readied for administration. The mice in the control and treatment groups received gavages of normal pig fecal suspension (10^8^ CFU/mL, 0.1 mL) and diarrheic pig fecal suspension (10^8^ CFU/mL, 0.1 mL), respectively, every two days until the end of the experiment. The results showed that the bodyweights of mice gavaged with diarrheic pig feces were significantly lower than those of the mice gavaged with normal pig feces (Figure 1A). In addition, the liver, spleen, and kidney indices of the mice gavaged with diarrheic pig feces were significantly increased (Figure 1B), and their colons were significantly shorter (Figure 1C). Immunofluorescence analysis revealed markedly more pronounced apoptosis in the colonic cells of the mice administered fecal material from diarrheic pigs via gavage (Figure 1D). In contrast, there were no significant differences regarding apoptosis in hepatic and ileal cells relative to the control group (Figure S1A,B). Western blot analysis indicated a significant increase in the apoptosis-related protein BAX, a significant decrease in the anti-apoptosis-related protein BCL2, and an increase in the cleavage activity of CASPASE-3 in colon tissues (Figure 1F). Proteins related to NRF2, namely, NRF2, HO-1, and NQO1, were significantly elevated in the treatment group (Figure 1G), suggesting activation of antioxidant defense pathways owing to oxidative stress. The DCLK1 protein was significantly downregulated in the treatment group (Figure 1H), indicating suppression of tuft cell activity. The upregulation of IRG1 indicated an enhanced immune response (Figure 1H), while the downregulation of SLC1A5 and SLC13A3 indicated reduced absorption of amino acids and itaconic acid by the body (Figure 1H). Immunofluorescence results showed a significant decrease in the expression of SLC1A5 in the colon (Figure 1E), with no significant changes in the ileum (Figure S1C). These results indicate that gavage of diarrheic pig feces leads to more severe colon damage.

### 3.2. Experiment 2: Itaconic Acid Alleviated the Adverse Effects of Diarrheic Pig Feces in Mice

**Figure 2:**
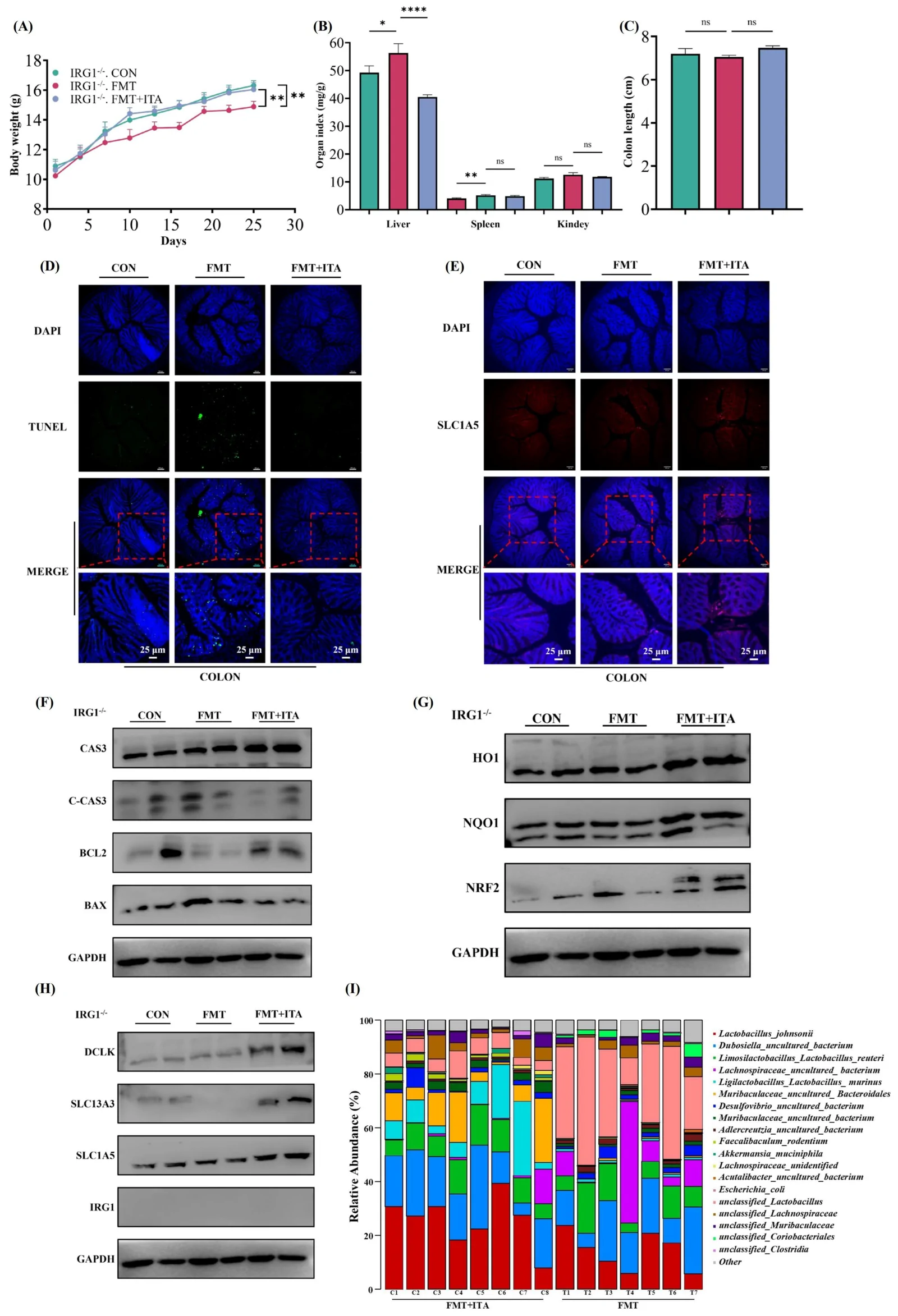
Itaconic acid alleviated the adverse effects of diarrheic pig feces in mice. (**A**) Daily weight gain of mice in the three groups: control group (gavaged with sterile normal saline), FMT group (gavaged with diarrheic pig feces), and FMT + itaconic acid group (gavaged with diarrheic pig feces + itaconic acid administered at 10 mg/kg). (**B**) Organ indices (liver, spleen, and kidney) of mice in the three groups. The organ index was calculated as follows: (organ weight/bodyweight). (**C**) Colon length of mice in the three groups. Colon samples were collected after sacrifice, and length was measured with a vernier caliper. (**D**) Immunofluorescence staining of cell apoptosis in the colon tissues. Tissues were stained with TUNEL reagent (green, apoptotic cells) and DAPI (blue, nuclear staining). Scale bar = 100 μm. (**E**) Immunostaining of the SLC1A5 transporter in colon tissues in the three groups. SLC1A5 was labeled with a specific primary antibody and a Cy3-conjugated secondary antibody (red), and nuclei were stained with DAPI (blue). Scale bar = 100 μm. (**F**) Expression of apoptosis-related proteins (BAX, BCL-2, and cleaved CASPASE-3) in colon tissues of mice in the three groups. (**G**) Expression of NRF2 pathway-related proteins in colon tissues of mice in the three groups. (**H**) Expression of itaconic acid-related proteins and amino acid transporter proteins in the colon tissues of mice in the control and treatment groups. (**I**) 16S rRNA gene sequencing analysis of fecal samples from the FMT group and FMT + itaconic acid group. All data are presented as means ± SEMs (n = 12 mice per group for (**A**–**C**); n = 6 biological replicates per group, with 5 fields per section for (**D**,**E**); n = 6 biological replicates per group for (**F**–**H**)). Statistical significance was determined via one-way analysis of variance (ANOVA) followed by Tukey’s post hoc test, where * *p* < 0.05, ** *p* < 0.01, **** *p* < 0.001 and ns (no significant difference) were considered statistically significant.

In Experiment 1, we found that levels of the IRG1 protein were significantly elevated in the group gavaged with diarrheic pig feces. To further explore the role of itaconic acid in this process, we used IRG1^−/−^ mice as research subjects. Four-week-old IRG1^−/−^ mice were evenly divided into three groups based on bodyweight: IRG1^−/−^ control (oral gavage of 0.2 mL of saline every other day), IRG1^−/−^ + FMT (oral gavage of 0.1 mL of diarrheic pig fecal suspension (10^8^ CFU/mL) supplemented with 0.1 mL of saline every other day to maintain an equal volume), and IRG1^−/−^ + FMT + ITA (administration of itaconate (ITA) via oral gavage at a dose of 10 mg/kg/day, plus oral gavage of diarrheic pig fecal suspension as performed in the IRG1^−/−^ + FMT group). The results showed that the mice in the IRG1^−/−^ + FMT group were significantly lighter than those in the other two groups (Figure 2A), and the liver and spleen indices were significantly higher. The liver index was the lowest in the IRG1^−/−^ + FMT + ITA group, while there was no significant difference in the kidney index among the three groups (Figure 2B). There was no significant difference in colon length among the three groups (Figure 2C). These results indicate that supplementation with itaconic acid can alleviate weight loss caused by gavage of diarrheic pig feces and reduce liver index values. Immunofluorescence observations revealed more severe diarrheic-pig-feces-induced colon cell apoptosis, which could be alleviated through the addition of itaconic acid (Figure 2D). But this situation was not obvious in the ileum and liver (Figure S1D,E). Western blot analysis showed a significant decrease in the apoptosis-related protein BAX, a significant increase in the anti-apoptosis-related protein BCL2, and a significant decrease in the cleavage activity of CASPASE-3 in the IRG1^−/−^ + FMT + ITA group (Figure 2F). The expression of NRF2, NQO1, and HO-1 proteins was significantly increased in the IRG1^−/−^ + FMT + ITA group, improving the antioxidant capacity of the mice (Figure 2G). Expression of DCLK1 was significantly increased, suggesting that itaconic acid may promote tuft cell proliferation (Figure 2H). Levels of proteins related to itaconic acid absorption, SLC13A3, and the glutamine absorption protein SLC1A5 were significantly higher after itaconic acid was added (Figure 2H). Immunofluorescence results showed a significant increase in the expression of SLC1A5 in the colon (Figure 2E), with no significant change in the ileum (Figure S1F). These results indicate that itaconic acid alleviates the adverse effects of diarrheic pig feces in mice.

### 3.3. Experiment 3: Multi-Omics Analysis of the Inhibition of Strain PCN033 by Itaconic Acid

**Figure 3:**
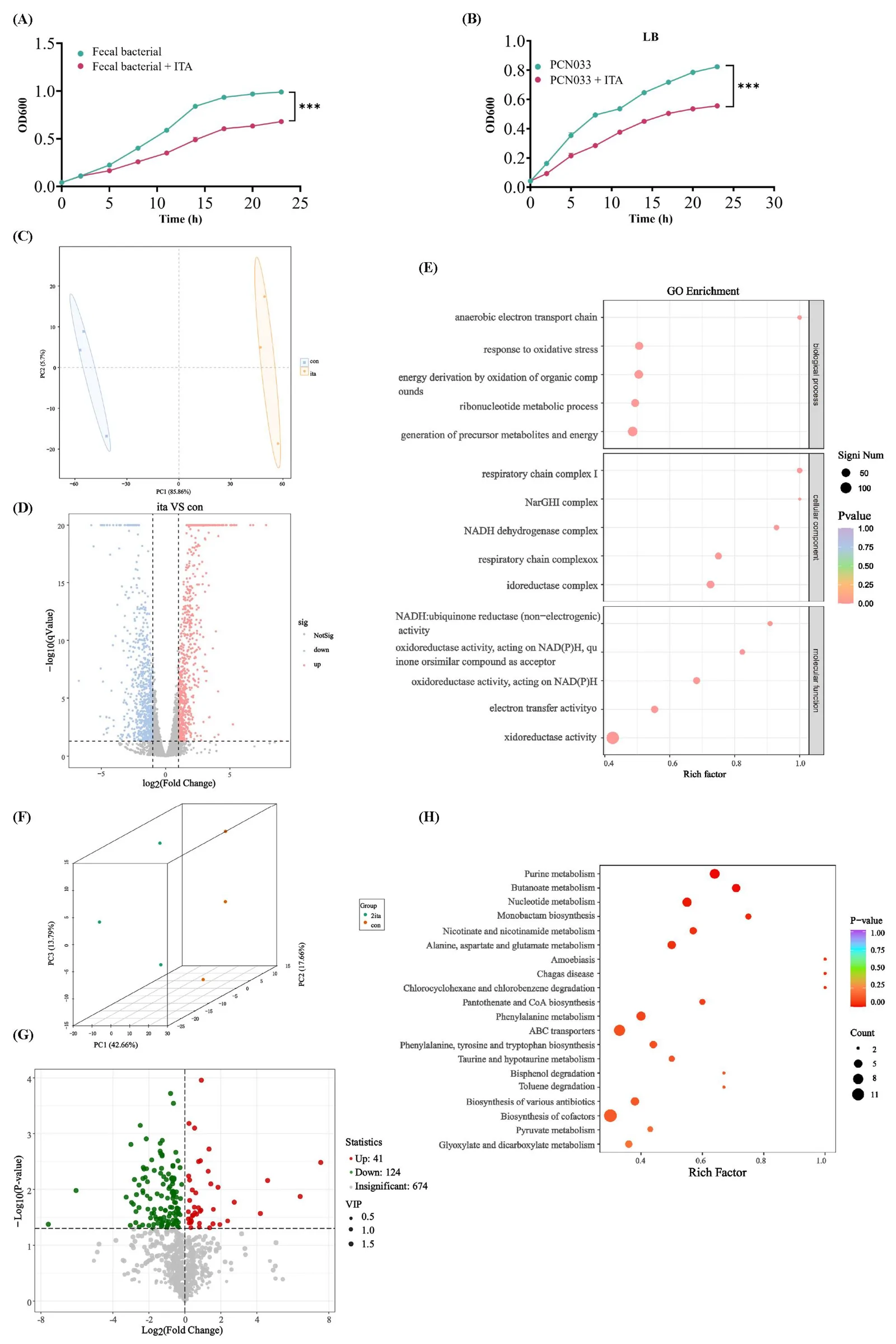
Multi-Omics Analysis of the Inhibition of Strain PCN033 by Itaconic Acid. (**A**) In vitro inhibitory effect of itaconic acid on the growth of microbes from diarrheic pig feces. (**B**) The inhibitory effect of itaconic acid on PCN033 growth. PCN033 was cultured in LB medium supplemented with itaconic acid. (**C**) PCA of the transcriptome in strain PCN033 with or without acid treatment. PCN033 was cultured in LB medium with or without 5 mM itaconic acid for 12 h, and total RNA was extracted for transcriptome sequencing. (**D**) Volcano plot of differentially expressed genes (DEGs) induced by itaconic acid in strain PCN033. (**E**) GO enrichment analysis of DEGs induced by itaconic acid in strain PCN033. (**F**) PCA of metabolites in the culture supernatant of strain PCN033 treated with or without itaconic acid. PCN033 was treated with or without 5 mM itaconic acid for 12 h, and culture supernatants were collected for LC-MS/MS analysis. (**G**) Volcano plot of differentially accumulated metabolites (DAMs) in the culture supernatant of strain PCN033 treated with or without itaconic acid. (**H**) GO enrichment analysis of DAMs in the culture supernatant of strain PCN033 treated with or without itaconic acid. All data are presented as means ± SEMs (n = 3). Statistical significance was determined via unpaired two-tailed Student’s *t*-test. *** *p* < 0.005 vs. the control group.

To further explore how itaconic acid alleviated diarrheic-pig-feces-gavage-induced weight loss in mice, we collected feces from the colons of mice from the three groups for 16S sequencing analysis. The results showed that itaconic acid significantly reduced the colonization of harmful bacteria from diarrheic pig feces in the intestine (Figure 2I and Figure S2A). In vitro experiments indicated that itaconic acid significantly inhibited the growth of microorganisms in the fecal microbiota (Figure 3A). We also found that itaconic acid inhibited the colonization of Escherichia coli in the gut. We selected the PCN033 strain to investigate the underlying mechanisms further. PCN033 is an Escherichia coli strain, originally isolated from a pig on a farm in our case, that is highly pathogenic and amenable to genetic editing. In vitro experiments showed that itaconic acid significantly inhibited the proliferation of PCN033 (Figure 3B). To elucidate the molecular mechanisms of this inhibition, we performed transcriptomic sequencing on PCN033. Principal component analysis (PCA) revealed a distinct separation between the ITA-treated and untreated groups (Figure 3C), and volcano plots indicated widespread alterations in gene expression following ITA treatment (Figure 3D). Gene Ontology (GO) enrichment analysis demonstrated that ITA disrupted the bacterial electron transport chain and exacerbated oxidative stress responses (Figure 3E). Furthermore, we conducted metabolomic analysis of ITA treatment. PCA revealed significant divergence in the metabolic profiles of the culture supernatant between the two groups (Figure 3F). Volcano plots highlighted substantial perturbations in metabolite abundance upon ITA addition (Figure 3G). Notably, GO enrichment analysis revealed that ITA severely impaired purine metabolism in PCN033 (Figure 3H).

### 3.4. Experiment 4: Itaconic Acid Can Directly Bind to the PurF Protein

**Figure 4:**
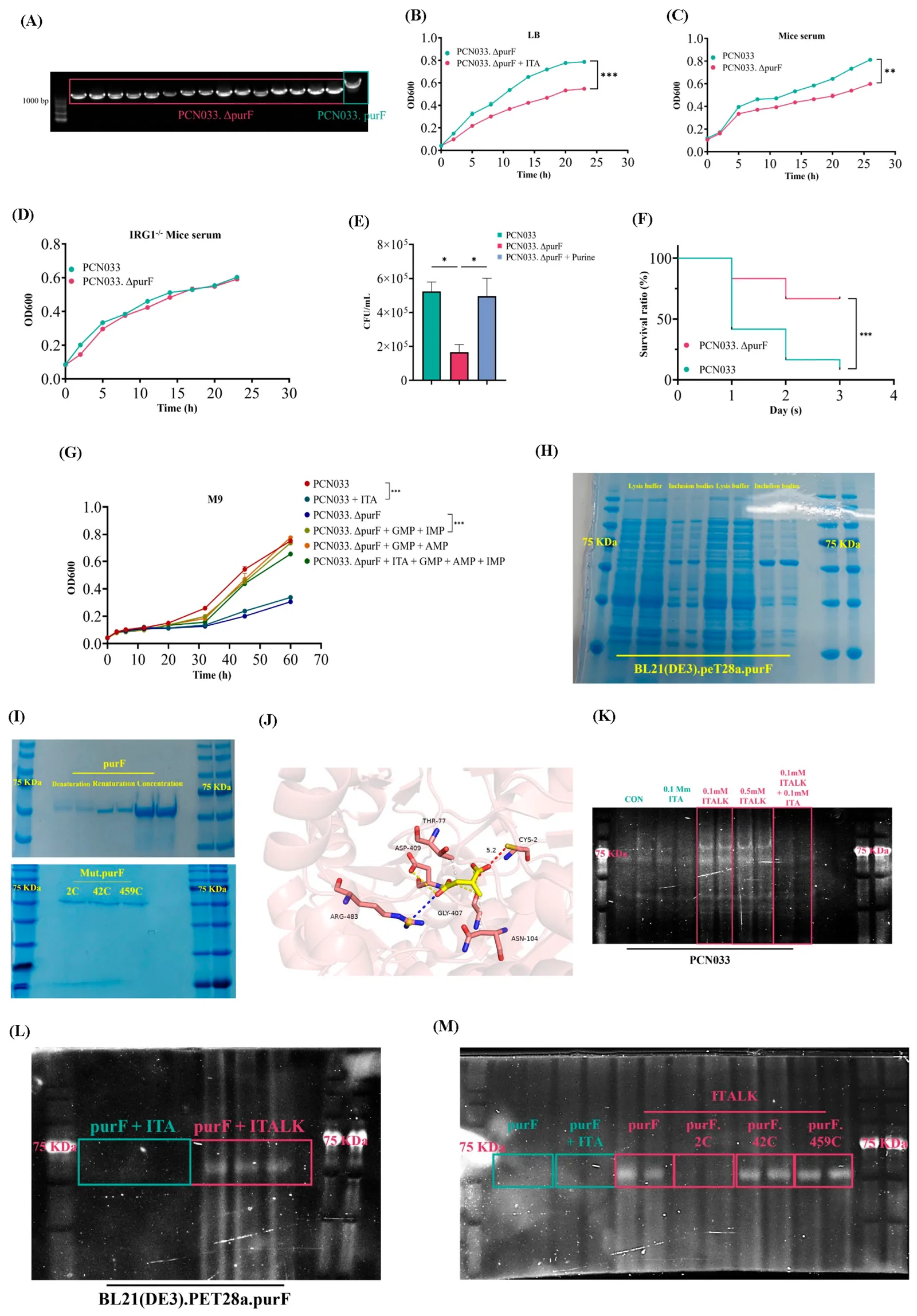
Itaconic acid can directly bind to the purF protein. (**A**) Construction and verification of purF knockout strain in PCN033 (PCN033.ΔpurF). (**B**) Inhibition of the growth of the PCN033.ΔpurF strain by itaconic acid. (**C**) Growth disadvantage of the PCN033.ΔpurF strain in normal serum. PCN033 and PCN033.ΔpurF were cultured in normal mouse serum, and bacterial growth was monitored by OD600. (**D**) Comparable growth of PCN033 and PCN033.ΔpurF strains in itaconate-depleted serum. PCN033 and PCN033.ΔpurF were cultured in itaconate-depleted mouse serum, and OD600 was measured. (**E**) The PCN033.ΔpurF strain exhibited increased susceptibility to cell killing, which was reversed by exogenous purine supplementation. Murine macrophages were co-cultured with PCN033 or PCN033.ΔpurF strain (MOI = 20:1) for 4 h, with or without 100 μM purine supplementation. (**F**) Lethality of PCN033 and PCN033.ΔpurF strains in acute infection assays. C57BL/6 mice were intraperitoneally infected with PCN033 or PCN033.ΔpurF. (**G**) Growth defect of the PCN033.ΔpurF strain in M9 minimal medium and the reversal of this defect by adenine and guanine supplementation. PCN033 and PCN033.ΔpurF were cultured in M9 minimal medium, with or without 100 μM adenine + guanine + inosine monophosphate. (**H**) Expression of the purF protein in different components. (**I**) Purification of PurF protein and construction of PurF point mutants. (**J**) Prediction of the itaconic acid binding site in PurF via molecular docking. Molecular docking model of PurF and itaconic acid, showing potential binding residues, along with binding energy calculations for the PurF–itaconic acid complex. Docking was performed using AutoDock Vina (version 1.2.5; The Scripps Research Institute). (**K**) Validation of itaconate–protein interaction in bacterial lysates via click chemistry. PCN033 lysates were incubated with azide-labeled itaconic acid and then subjected to a click reaction with alkyne-rhodamine. (**L**) Verification of direct binding between PurF and itaconic acid via click chemistry. Purified PurF protein was incubated with azide-labeled itaconic acid and then subjected to a click reaction with alkyne-rhodamine. (**M**) Identification of the precise itaconic-acid-binding residues in PurF via click chemistry. PurF point mutants (2C, 42C, and 459C) were purified and incubated with azide-labeled itaconic acid. Click reactions were performed to detect binding. All data are presented as means ± SEMs (n = 3 biological replicates for (**A**–**E**,**G**,**K**–**M**); n = 10 mice per group for (**F**)). Statistical significance was determined via one-way analysis of variance (ANOVA) followed by Tukey’s post hoc test, where * *p* < 0.05, ** *p* < 0.01 and *** *p* < 0.005 were considered statistically significant.

Purine metabolism is critical for the survival and proliferation of *E. coli*, and an intact de novo purine synthesis pathway is essential for the independent survival of *E. coli* in a minimal medium. To investigate whether itaconic acid directly inhibits specific proteins in purine metabolism, we selected PRPP amidotransferase (purF) for gene knockout (Figure 4A). Following gene knockout, the proliferation of PCN033.ΔpurF significantly decreased after itaconic acid was added (Figure 4B). Relative to the normal bacteria, the knockout bacteria lacked a growth advantage in normal mouse serum (Figure 4C), but this growth disadvantage was compensated for in IRG1-deficient serum (Figure 4D). Our co-culture experiments showed that the knockout bacteria had a lower survival rate when co-cultured with the macrophage Raw264.7, and this survival disadvantage could be compensated for by exogenous supplementation with purines (Figure 4E). In M9 minimal medium, the knockout bacteria’s growth disadvantage was more pronounced, and it could be compensated for by exogenous supplementation with adenine and guanine (Figure 4G). Mouse challenge experiments showed that the knockout bacteria led to a reduced mortality rate among the mice (Figure 4F), suggesting that itaconic acid may exert antibacterial effects by inhibiting purine metabolism. To further investigate the binding sites of itaconic acid and purF, we expressed purF in vitro and found that the purF protein formed inclusion bodies in the BL21(DE3) vector (Figure 4H). We dissolved, purified, and concentrated the inclusion bodies via chemical denaturation (Figure 4I). Using molecular docking, which predicted that itaconic acid might bind to cysteine (Figure 4J), we constructed three cysteine mutants of purF at positions 2C, 42C, and 459C (Figure 4I). Click chemistry experiments showed that ITALK bound to proteins in the BL21(DE3) lysate, and this binding was competitive with itaconic acid. The bonding effect weakened after a high concentration of itaconic acid was added (Figure 4K). We then observed that ITALK could bind to purified purF (Figure 4L). When ITALK was subjected to click chemistry reactions with the three mutants, we found that the binding effect of ITLAK and purF disappeared only after the 2C mutation, suggesting that the second cysteine is an important itaconic acid binding site in purF (Figure 4M).

### 3.5. Experiment 5: Itaconic Acid Can Alleviate Adverse Reactions Caused by the PCN033.ΔpurF in Nursery Pigs

**Figure 5:**
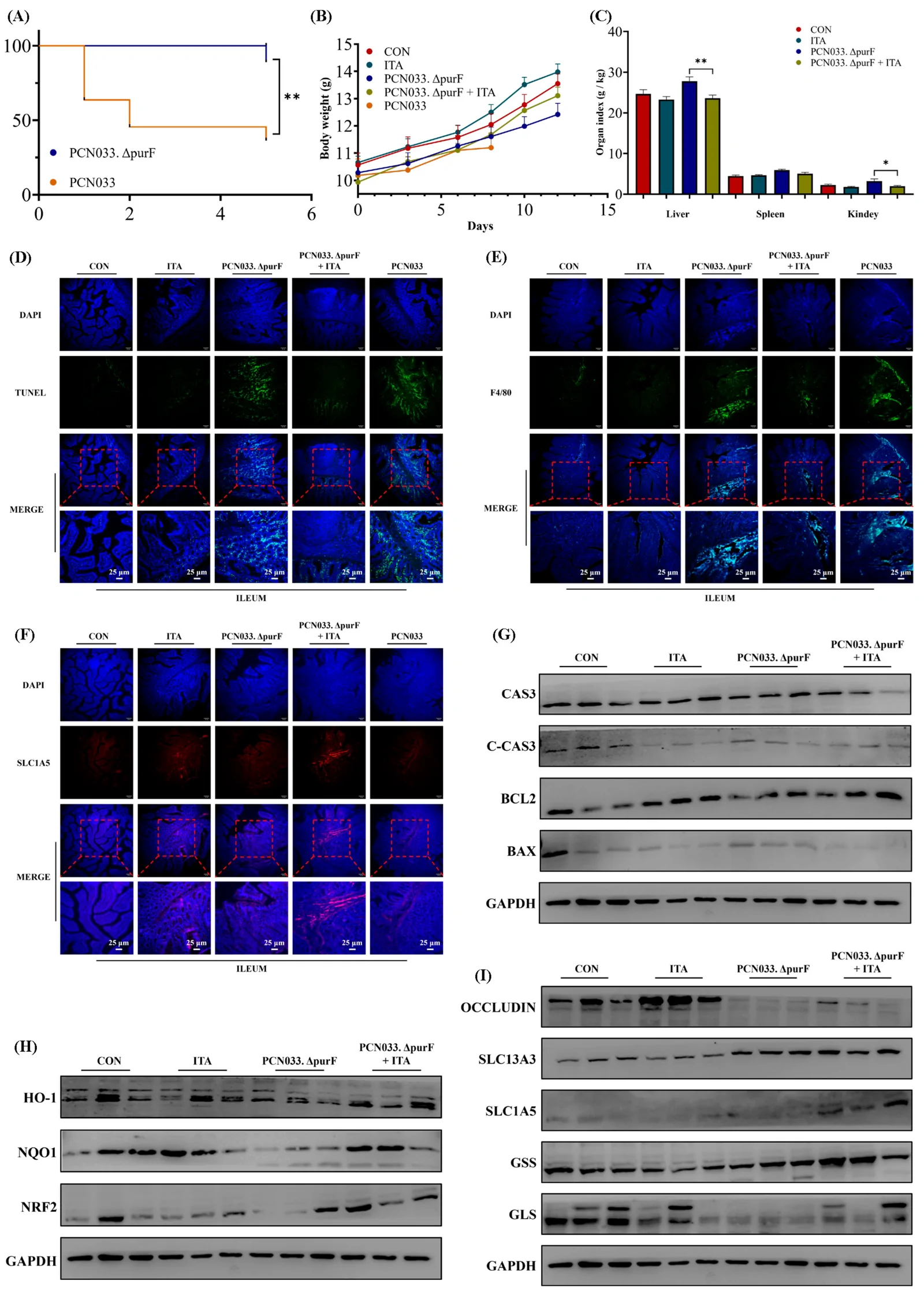
Itaconic acid alleviates adverse reactions caused by PCN033.ΔpurF in nursery pigs. (**A**) Mortality rates in different nursery pig treatment groups: the HV group (PCN033) and the LV group (PCN033.ΔpurF). (**B**) Daily weight gain of nursery pigs in different treatment groups. (**C**) Organ indices (liver, spleen, and kidney) of nursery pigs in different treatment groups. The organ index was calculated as follows: (organ weight/bodyweight). (**D**) Immunofluorescence staining of cell apoptosis in the colon tissues. Tissues were stained with TUNEL reagent (green, apoptotic cells) and DAPI (blue, nuclear staining). Scale bar = 100 μm. (**E**) Immunofluorescence staining of F4/80 in the ileum tissues of nursery pigs in different treatment groups. F4/80 (macrophage marker) was labeled with a specific primary antibody and a FITC-conjugated secondary antibody (green), and nuclei were stained with DAPI (blue). Scale bar = 100 μm. (**F**) Immunofluorescence staining of SLC1A5 in the ileum tissues of nursery pigs in different treatment groups. SLC1A5 was labeled with a specific primary antibody and a Cy3-conjugated secondary antibody (red), and nuclei were stained with DAPI (blue). Scale bar = 100 μm. (**G**) Expression of apoptosis-related proteins (BAX, BCL-2, and cleaved CASPASE-3) in the ileum tissues of nursery pigs in different treatment groups. (**H**) Expression of NRF2 pathway-related proteins (NRF2, HO-1 and NQO1) in the ileum tissues of nursery pigs in different treatment groups. (**I**) Expression of itaconic acid transport and glutamine-metabolism-related proteins in different treatment groups. All data are presented as means ± SEMs (n = 7–12 pigs per group for (**A**–**C**); n = 6 biological replicates per group, randomly selected from each group for (**G**–**I**); n = 6 biological replicates per group, 5 fields per section for (**D**–**F**)). Statistical significance was determined via one-way analysis of variance (ANOVA) followed by Tukey’s post hoc test, where * *p* < 0.05 and ** *p* < 0.01 were considered statistically significant.

We conducted experiments on the inhibition of PCN033 by itaconic acid in nursery pigs. Weanling piglets were randomly divided into five groups: (1) control (CON), (2) ITA (itaconate at 10 mg/kg/day in feed), (3) PCN033.ΔpurF (low-virulence challenge (LV)), (4) PCN033.ΔpurF + ITA (LV + ITA), and (5) PCN033 (high-virulence challenge (HV)). After pre-feeding, the pigs were subjected to an intraperitoneal challenge with a dose of 10^8^ CFU/mL in 10 mL every three days until the end of the experiment. As shown, the HV caused consecutive deaths during the challenge period, while the LV group maintained a low mortality rate after the challenge (Figure 5A). Relative to the CON group, the mice in the challenge groups became lighter, and the addition of itaconic acid had a partial alleviating effect (Figure 5B). Organ indices showed that the liver and kidney indices of the HV group were significantly increased, and the addition of itaconic acid had a potent alleviating effect (Figure 5C). Immunofluorescence staining showed that ileal cell apoptosis was most pronounced in the HV and LV groups (Figure 5D), and apoptosis also occurred in the liver and colon (Figure S3A,B). The addition of itaconic acid alleviated this phenotype. The status of inflammatory cell infiltration (F4/80) showed that there was obvious inflammatory infiltration in the HV and LV groups, and the addition of itaconic acid alleviated this situation (Figure 5E). Western blot analysis indicated that the addition of itaconic acid promoted the expression of the anti-apoptotic protein BCL2, inhibited the expression of the pro-apoptotic protein BAX, and inhibited the cleavage activity of CASPASE-3 (Figure 5G). The addition of itaconic acid significantly increased the expression of NRF2, NQO1, and HO-1, improving antioxidant performance (Figure 5H). Under challenge conditions, the addition of itaconic acid significantly promoted the absorption and metabolism of itaconic acid and glutamine (Figure 5I). Immunofluorescence results showed that SLC1A5 promoted glutamine absorption after the addition of itaconic acid (Figure 5F). In conclusion, the addition of itaconic acid can alleviate the adverse reactions caused by a challenge with the PCN033 knockout strain.

## 4. Discussion

Post-weanling diarrhea is well known to impair nutrient digestion, absorption, metabolism, and intestinal barrier function in piglets, resulting in substantial economic losses in the swine industry. Previous studies have confirmed that proliferation of pathogenic *Escherichia coli* (*E. coli*) is closely associated with post-weanling diarrhea, yet effective therapeutic strategies remain scarce. Notably, there is a paucity of research regarding whether itaconic acid can be used as a feed additive to mitigate weaning stress or bacterial infections in piglets, and this research gap serves as the core motivation for this study.

Itaconic acid, a naturally occurring organic acid, has been shown to exhibit antibacterial properties, primarily by suppressing the bacterial respiratory chain to inhibit Salmonella proliferation and regulating bacterial biofilm formation [26,27,28]. However, its regulatory effects on pathogenic *E. coli*—especially highly pathogenic strains such as PCN033, which is closely linked to piglet post-weanling diarrhea—remain unclear. Furthermore, the potential molecular mechanisms underlying itaconic acid’s antibacterial activity against pathogenic *E. coli* and its practical application value in swine production have not been systematically explored.

To address these research gaps, we designed a series of experiments to clarify the role of itaconic acid in alleviating pathogenic *E. coli*-induced disorders in piglets and identify its specific molecular targets. First, we transplanted fecal microbiota from diarrheic pigs into healthy mice and administered itaconic acid (experiments 1 and 2) to investigate its beneficial effect. As expected, we found that gavage of feces from diarrheic pigs led to weight loss in mice, accompanied by increased colonization of harmful bacteria. These effects were significantly alleviated by itaconic acid administration. These in vivo findings confirmed that itaconic acid can mitigate diarrhea-related adverse reactions.

Next, we confirmed that itaconic acid can inhibit the proliferation of PCN033 in vitro, and then multi-omics approaches were used to explore specific molecular targets in our animal model. Notably, in rescue experiments related to itaconic acid, 4-octyl itaconate (4-OI, a cell-penetrating derivative of itaconic acid) is typically chosen because of findings showing that 4-OI supplementation increased survival rates and reduced inflammation in response to lipopolysaccharide [29] and dextran sulfate sodium treatment in mice, respectively [30]. In our study, itaconic acid, rather than 4-OI, was used, as 4-OI is costly and unsuitable for large-scale production applications. While it is well established that orally administered free ITA is hindered by challenges such as rapid absorption and subsequent hepatic and renal metabolism, recent evidence has clarified its physiological relevance in the gut [31]. Studies have demonstrated that dietary ITA can successfully reach the distal intestine (e.g., the cecum and colon) and perform significant biological functions [31,32,33,34]. Furthermore, ITA’s antimicrobial efficacy is highly pH-dependent; in the acidic microenvironment of the gut, its antibacterial activity is substantially enhanced [35,36]. Therefore, even if the local luminal concentration of ITA falls below the in vitro inhibitory threshold, the acidic conditions in the distal gut can potentiate its antimicrobial effects, thereby validating our decision to use free ITA in our in vivo model.

It has been reported that certain bacteria possess itaconic acid binding sites, and the binding of itaconic acid triggers subsequent response elements for its decomposition [37,38]. Accordingly, we wanted to know whether PCN033 also contains itaconic-acid-binding proteins. We found that the transcriptome proteins upregulated or downregulated by itaconic acid were primarily involved in immune responses, antioxidant responses, and ribosomal metabolic processes. We performed NCBI BLAST+ suite (version 2.16.0; National Center for Biotechnology Information, Bethesda, MD, USA) analysis by comparing the differentially expressed genes in the transcriptome with known itaconic-acid-binding proteins. Unexpectedly, we did not identify direct itaconic acid binding sites in *E. coli* PCN033 using the BLAST analysis, with a similarity rate of no more than 20%. In addition to direct binding, itaconic acid can also regulate protein function through protein modification or by regulating [39] purine metabolism of bacteria. In this study, metabolome sequencing of PCN033 indicated that itaconic acid significantly inhibited purine metabolism—a crucial bioprocess for bacterial survival and proliferation—a finding that is in agreement with a previous study [40]. PRPP amidotransferase (PurF) is a key enzyme in de novo purine synthesis. To determine whether itaconic acid could bind to PurF and contribute to the effect observed, a molecular docking model assay was conducted. We found that itaconic acid can form covalent bonds with the active pocket of PurF, verifying the possibility of direct binding between itaconic acid and PurF. Given the challenges associated with determining itaconic acid levels in biological systems, such as low sensitivity, susceptibility to background interference, and dynamic-tracking difficulties [41], we utilized a labeled itaconic acid derivative, ITALK (itaconate-alkyne). ITALK can covalently bind to proteins through click chemistry reactions, forming stable covalent bonds, and is fluorescently labeled for subsequent detection, explaining its use in previous studies [39,42]. Our click chemistry results demonstrate that itaconic acid can bind to PurF, as shown in Figure 4K. Although PCN033 serves as a well-established and highly pathogenic model for academic research, specific mortality data following infection of pig herds with this strain remain limited. To solidify our data in the swine sector, we constructed a purF knockout strain and administered the wild-type or mutant strain to piglets in the presence or absence of itaconic acid. As expected, we found that knocking out the purF gene significantly reduced the overall lethality of PCN033, and itaconic acid effectively alleviated weight loss, diarrhea, and vomiting induced by the PCN033 purF knockout strain. These data indicate that purF is a key virulence factor for PCN033 infection and is critical for the development of novel vaccines against pathogenic *E. coli*.

## 5. Conclusions

In conclusion, our in vitro and in vivo findings demonstrate that itaconic acid effectively inhibits the growth and pathogenicity of *Escherichia coli* strain PCN033 and alleviates bacterial diarrhea in animals. Mechanistically, itaconic acid targets purine metabolism via covalent binding to PurF, thereby exerting its antimicrobial effects. Itaconic acid administration may be suitable for use as a therapeutic strategy for alleviating PCN033-infection-induced intestinal barrier dysfunction by targeting bacterial purine metabolism, thereby benefiting animal health and sustainable development.

## Data Availability

The dataset is publicly accessible without embargoes or login restrictions under a CC-BY license. The raw data supporting the conclusions of this article have been deposited in Figshare and are publicly available under the following DOI: 10.6084/m9.figshare.32625699.

## Supplementary Materials

The following supporting information can be downloaded at: https://www.mdpi.com/article/10.3390/microorganisms14081852/s1. Figure S1: Immunofluorescence analysis of apoptosis and SLC1A5 expression in different tissues. Figure S2: Principal component analysis (PCA) of the 16S sequencing data. Figure S3: Immunofluorescence analysis of macrophage infiltration in liver and colon tissues.

## Abbreviations

ITA	Itaconic acid
PurF	Phosphoribosyl pyrophosphate amidotransferase
ExPEC	Porcine extraintestinal pathogenic Escherichia coli
PCN033	Escherichia coli PCN033
DNPS	De novo purine synthesis
IMP	Inosine monophosphate
AMP	Adenosine monophosphate
GMP	Guanosine monophosphate
FMT	Fecal microbiota transplantation
SFF	Sterile fecal filtrate
IRG1	Iron-responsive gene 1
APRT	Adenine phosphoribosyltransferase
ITALK	Itaconate-alkyne
4-OI	4-Octyl itaconate
