# Supplementary material for: Itaconic Acid Directly Binds to the Cysteine Residue of PurF: A Novel Mechanism for Inhibiting Purine Metabolism in Porcine Pathogenic Escherichia coli
Source: Microorganisms. 2026 Aug 20;14(8):1852. doi: 10.3390/microorganisms14081852 (PMC13515712; doi:10.3390/microorganisms14081852)
Supplement: Supplementary file 1 [file microorganisms-14-01852-s001.zip › microorganisms-4451528-supplementary.pdf]

Supplementary materials

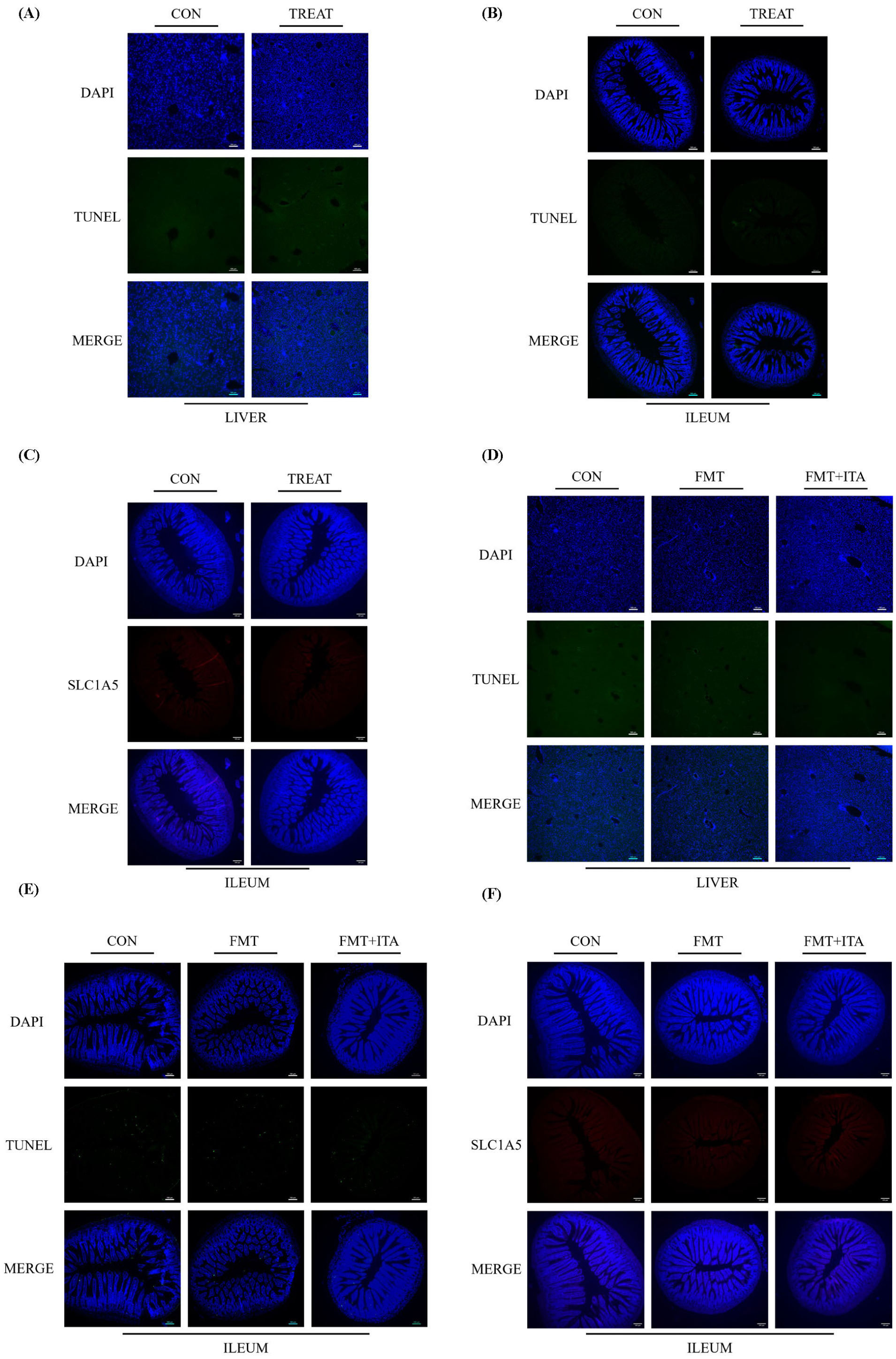

Figure S1

(A) Immunofluorescence co-localization of TUNEL (green) and DAPI (blue) in liver tissues of CON and TREAT groups. TUNEL-positive apoptotic cells are labeled green,

and cell nuclei are counterstained with DAPI (blue). Scale bar = 100 μm. (B) Immunofluorescence co-localization of TUNEL (green) and DAPI (blue) in ileum tissues of CON and TREAT groups. TUNEL-positive apoptotic cells are labeled green, and cell nuclei are counterstained with DAPI (blue). Scale bar = 100 μm. (C) Immunofluorescence analysis of SLC1A5 (red) and DAPI (blue) in ileum tissues of CON and TREAT groups. SLC1A5 (amino acid transporter) is labeled red, and cell nuclei are counterstained with DAPI (blue). Scale bar = 100 μm. (D) Immunofluorescence co-localization of TUNEL (green) and DAPI (blue) in liver tissues of CON, FMT, and FMT+ITA groups. TUNEL-positive apoptotic cells are labeled green, and cell nuclei are counterstained with DAPI (blue). Scale bar = 100 μm. (E) Immunofluorescence co-localization of TUNEL (green) and DAPI (blue) in ileum tissues of CON, FMT, and FMT+ITA groups. TUNEL-positive apoptotic cells are labeled green, and cell nuclei are counterstained with DAPI (blue). Scale bar = 100 μm. (F) Immunofluorescence analysis of SLC1A5 (red) and DAPI (blue) in ileum tissues of CON, FMT, and FMT+ITA groups. SLC1A5 is labeled red, and cell nuclei are counterstained with DAPI (blue). Scale bar = 100 μm. All data are presented as mean ± SEM (n = 6 mice per group, 5 fields per section). Statistical significance was determined by unpaired two-tailed Student's t-test. *P < 0.05, **P < 0.01 vs. control group.

(A)

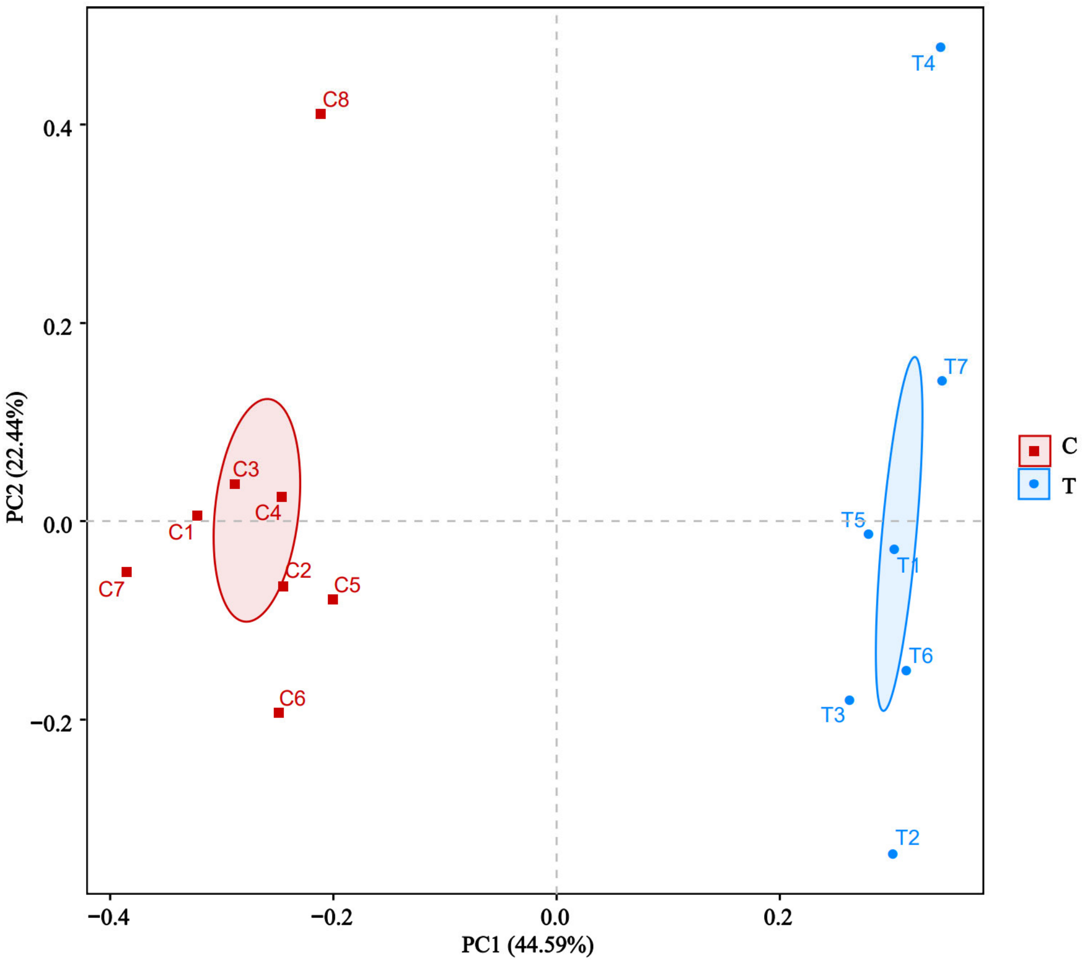

Figure S2

(A) Principal component analysis (PCA) of the 16S sequencing data was performed to compare the CON group and the treatment group.

(A)

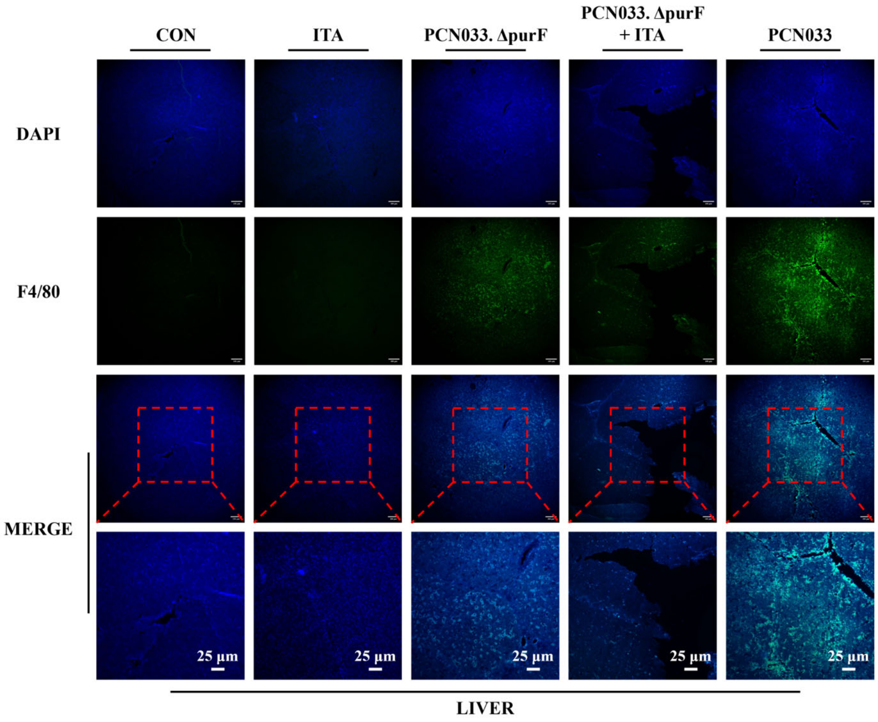

(B)

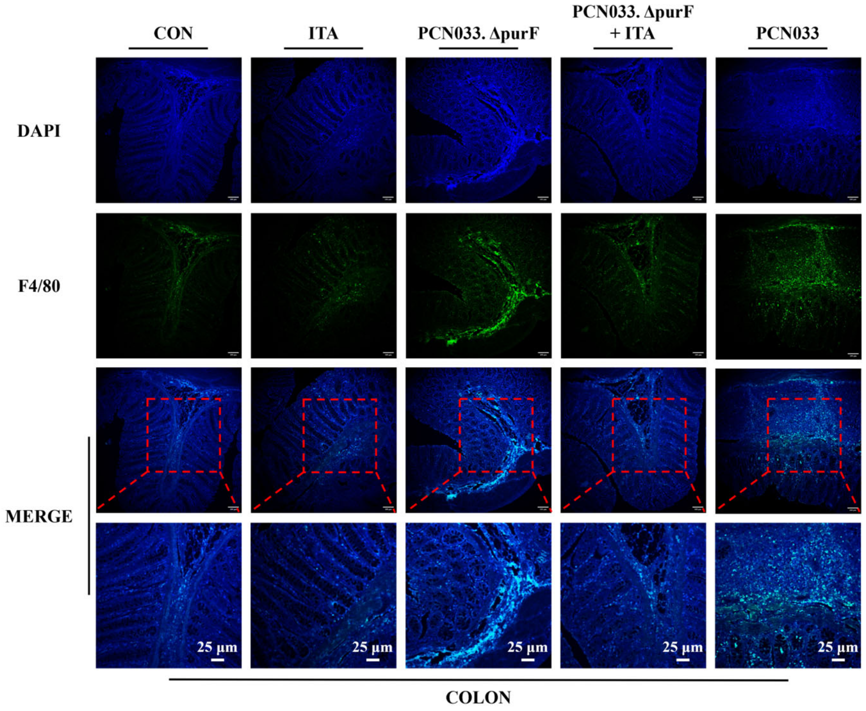

Figure S3

(A) Immunofluorescence co-localization of F4/80 (green, macrophage marker) and DAPI (blue) in liver tissues of CON, ITA, PCN033.ΔpurF, PCN033.ΔpurF + ITA, and PCN033 groups. F4/80-positive macrophages are labeled green, and cell nuclei are

counterstained with DAPI (blue). The red dashed box indicates the magnified view of the selected region. Scale bar = 100 μm. (B) Immunofluorescence co-localization of F4/80 (green, macrophage marker) and DAPI (blue) in colon tissues of CON, ITA, PCN033.ΔpurF, PCN033.ΔpurF + ITA, and PCN033 groups. F4/80-positive macrophages are labeled green, and cell nuclei are counterstained with DAPI (blue). The red dashed box indicates the magnified view of the selected region. Scale bar = 100 μm. All data are presented as mean ± SEM (n = 6 pigs per group, 5 fields per section). Statistical significance was determined by unpaired two-tailed Student's t-test. *$P < 0.05$, **$P < 0.01$ vs. control group.
